# Hyperglycemia Aggravates Periodontitis via Autophagy Impairment and ROS-Inflammasome-Mediated Macrophage Pyroptosis

**DOI:** 10.3390/ijms24076309

**Published:** 2023-03-27

**Authors:** Zhenxing Zhao, Ye Ming, Xiang Li, Hao Tan, Xinyi He, Lan Yang, Jinlin Song, Leilei Zheng

**Affiliations:** 1 College of Stomatology, Chongqing Medical University, Chongqing 401147, China; 2 Chongqing Key Laboratory of Oral Diseases and Biomedical Sciences, Chongqing 401147, China; 3 Chongqing Municipal Key Laboratory of Oral Biomedical Engineering of Higher Education, Chongqing 401147, China

**Keywords:** hyperglycemia, periodontal disease, pyroptosis, MCC950, oxidative stress, autophagy

## Abstract

Macrophage pyroptosis drives the secretion of IL-1β, which has been recently reported to be a featured salivary biomarker for discriminating periodontitis in the presence of diabetes. This study aimed to explore whether macrophage pyroptosis plays a role in the development of diabetes mellitus–periodontitis, as well as potential therapeutic strategies. By establishing a model of experimental diabetes mellitus–periodontitis in rats, we found that IL-1β and gasdermin D were highly expressed, leading to aggravated destruction of periodontal tissue. MCC950, a potent and selective molecule inhibitor of the NLRP3 inflammasome, effectively inhibited macrophage pyroptosis and attenuated alveolar bone losses in diabetes mellitus–periodontitis. Consistently, in vitro, high glucose could induce macrophage pyroptosis and thus promoted IL-1β production in macrophages stimulated by lipopolysaccharide. In addition, autophagy blockade by high glucose via the mTOR-ULK1 pathway led to severe oxidative stress response in macrophages stimulated by lipopolysaccharide. Activation of autophagy by rapamycin, clearance of mitochondrial ROS by mitoTEMPO, and inhibition of inflammasome by MCC950 could significantly reduce macrophage pyroptosis and IL-1β secretion. Our study demonstrates that hyperglycemia promotes IL-1β production and pyroptosis in macrophages suffered by periodontal microbial stimuli. Modulation of autophagy activity and specific targeting of the ROS-inflammasome pathway may offer promising therapeutic strategies to alleviate diabetes mellitus–periodontitis.

## 1. Introduction

Diabetes mellitus and periodontitis are both common and chronic diseases. Findings from multiple studies demonstrate that diabetes mellitus leads to a hyperinflammatory response to the periodontal microbiota, which leads to an increase in inflammatory cytokine expression and aggravated destruction of periodontal tissue [1]. Macrophages, the critical innate immune cells in periodontal tissue, are responsible for phagocytosis and digestion of microorganisms, activation of adaptive immunity, inflammatory response, as well as tissue repair [2,3]. However, diabetes mellitus increases the production of inflammatory cytokines in macrophages, which remain in an inflammatory state and fail to transition to a reparative phenotype [4]. It has been reported that the gingival crevicular fluid and monocytic secretion of IL-1β was significantly higher in diabetics compared with non-diabetic controls. This difference could be due to hyperinflammatory response to the microbiota in periodontal tissue, as a consequence of macrophage under hyperglycemia.

Pyroptosis is an inflammatory form of programmed cell death implicated in both immune protection and pathological inflammation from overexuberant immune response to infections [5]. The protein GSDMD is a key executioner of pyroptosis, which forms pores in the plasma membrane of cells and thus results in the release of inflammatory cytokine, such as IL-1β and IL-18 [6,7]. The NLRP3/caspase-1/GSDMD pathway leads to pyroptosis and is significantly related to inflammation. Pyroptosis has been recently suggested as one of the pathways implicated in the pathogenesis of periodontitis [8,9]. In addition, hyperglycemia, the hallmark of diabetes mellitus, impairs the function of macrophage and induces pyroptosis, which contributes to cell senescence and low-degree inflammatory state in periodontal tissue [10]. The critical role of macrophage pyroptosis in periodontitis or diabetes mellitus has sparked intense interest, but little is known about how macrophage pyroptosis influences diabetes mellitus-associated periodontitis.

MCC950 is a diarylsulfonylurea-containing compound and selective inhibitor of the NLRP3 inflammasome. MCC950 specifically inhibits the NLRP3 inflammasome but not the major anti-microbial inflammasomes NLRP1 or NLRC4, and MCC950 may therefore have less immunosuppressive effects [11]. In addition, the effect of MCC950 has been verified in various animal models, such as cerebral ischemia [12], atherosclerosis [13], aortic aneurysms [14], cholestatic liver injury [15], and acute pancreatitis [16]. As yet, pharmacological blockade of the inflammasome and pyroptosis by MCC950 has not been investigated in diabetes mellitus–periodontitis. The aim of this study was to explore the role of macrophage pyroptosis in the development of diabetes mellitus–periodontitis by establishing an animal model, as well as the effect of MCC950 on diabetes mellitus–periodontitis. Combined with an in vitro model, we aimed to investigate the molecular mechanism inducing macrophage pyroptosis in order to provide specific targets for the treatment and prevention of diabetes mellitus–periodontitis.

In the present study, for the first time, we established rat models to investigate the role of macrophage pyroptosis in the process of diabetes mellitus–periodontitis. To mimic the hyperglycemia and pro-inflammatory conditions in vivo, THP-1 and U937 macrophages treated with high glucose and lipopolysaccharide (LPS) were used to explore the role of autophagy and oxidative stress in the process of pyroptosis. Our study demonstrates that hyperglycemia impairs autophagy, leads to the accumulation of dysfunctional ROS-generating mitochondria in macrophages stimulated by LPS, and induces activation of inflammasome, which promotes macrophage pyroptosis and aggravates periodontitis.

## 2. Results

### 2.1. Aberrant Activation of Macrophage Pyroptosis in Diabetes Mellitus–Periodontitis

**Figure 1:**
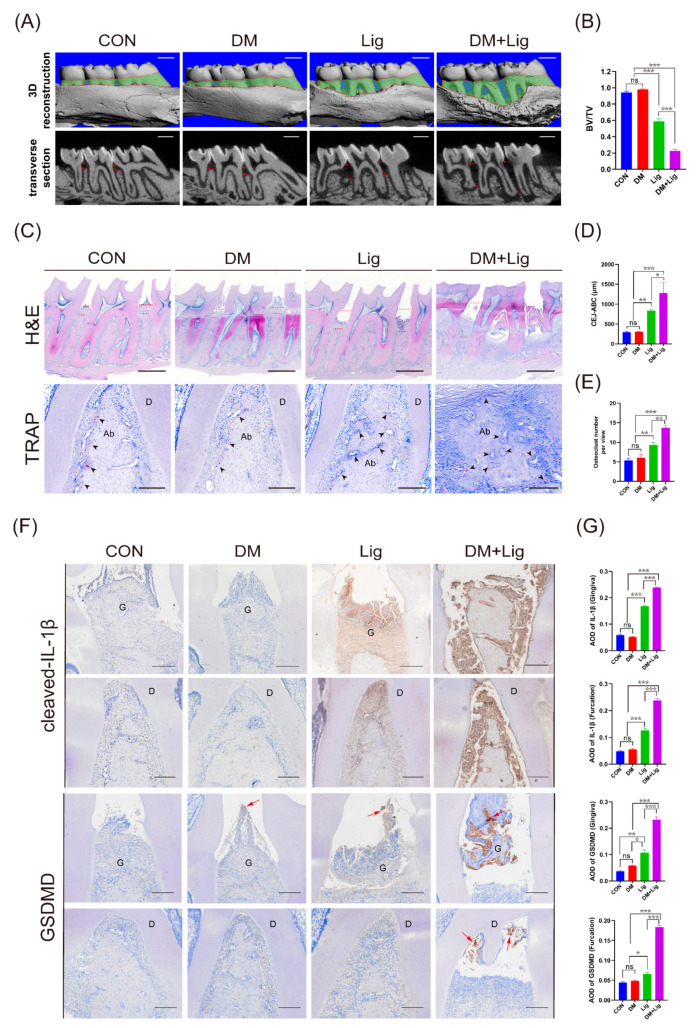

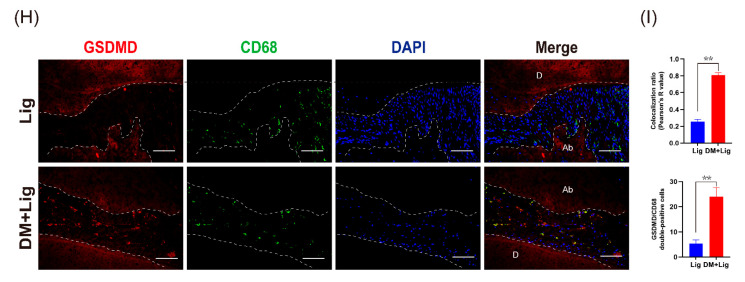
Diabetes mellitus–periodontitis presented exacerbated bone resorption and aberrant activation of macrophage pyroptosis. (**A**) Representative 3D reconstructions and transverse section images of control (CON), diabetes mellitus (DM), ligature-induced periodontitis (Lig), and DM + Lig groups. The green area displays the exposure of the root (scale bar: 1 mm). (**B**) Bone volume/total volume (BV/TV). (**C**) Representative images of HE staining (upper panel) in coronal sections of periodontal tissue (scale bar: 1 mm). Representative images of TRAP staining (lower panel) in the furcation region of the second molar (scale bar: 200 μm). (**D**) Distances between the CEJ and the ABC. (**E**) Quantitative analysis of osteoclast number at the alveolar bone. (**F**) Immunohistochemistry staining against IL-1β and GSDMD of periodontal tissues (scale bar: 200 μm). Red arrows demonstrate GSDMD positive cells. (**G**) Quantitative analysis of IL-1β and GSDMD (total GSDMD and cleaved GSDMD) in the gingiva and the furcation of the second molar. (**H**) Representative images of immunofluorescence staining of macrophage marker (CD68) and pyroptosis marker (GSDMD) in the periodontium of the Lig group and the DM + Lig group. Nuclei were stained with DAPI (blue). Scale bar, 100 μm. (**I**) Pearson’s R value shows the degree of co-localization of GSDMD and CD68 determined using ImageJ software. The number of GSDMD/CD68 double-stained cells was analyzed. Data in (**B**,**D**,**E**,**G**,**I**) are shown as mean ± SD (*n* = 4). * *p* < 0.05. ** *p* < 0.01. *** *p* < 0.001. Ab, alveolar bone; G, gingiva; D, dentin; DM, diabetes mellitus; CEJ-ABC, cementoenamel junction and alveolar bone crest; AOD, average optical density.

To investigate the influence of diabetes mellitus on periodontitis, SD rats were first rendered hyperglycemic following high-fat diet and injections of streptozocin (STZ), and then sterile silk ligatures were placed at the cervical areas of maxillary second molars to induce periodontitis (Figure S1A,B). Three-dimensional reconstructions of maxilla from rats in the DM + Lig group exhibited exacerbated alveolar bone loss than rats in the Lig group (Figure 1A). HE staining also showed severe alveolar bone loss and much more increase in the cementoenamel junction and alveolar bone crest (CEJ-ABC) distance in the DM + Lig group compared with those in the other three groups (Figure 1C,D). There was a significant decrease in BV/TV in the furcation region of the DM + Lig group (Figure 1B). By TRAP staining, higher amounts of TRAP+ cells could be observed in the furcation region of the DM + Lig group compared with that of the Lig group (Figure 1C,E). Moreover, immunohistochemistry staining revealed that the expression of IL-1β and GSDMD was significantly higher in the periodontal tissue of rats in the DM + Lig group than rats in the other three groups (Figure 1F,G). Noteworthily, GSDMD+ cells could be observed in the gingiva of rats in the DM group, but were less than the Lig group and the DM + Lig group. In the periodontal tissue, IL-1β is mainly expressed by macrophages and dendritic cells. Immunofluorescence staining showed amounts of CD68+ macrophages infiltrated in periodontic lesions of the Lig and DM + Lig groups. In addition, we can observe increased colocalization of CD68+ and GSDMD+ cells in the periodontium of the DM + Lig group, which indicated aberrant macrophage infiltration and pyroptosis (Figure 1H,I). These collective results showed that IL-1β was at high expression in diabetes mellitus–periodontitis due to macrophage pyroptosis.

### 2.2. MCC950 Attenuates Destruction of Periodontal Tissue in Diabetes Mellitus–Periodontitis

**Figure 2:**
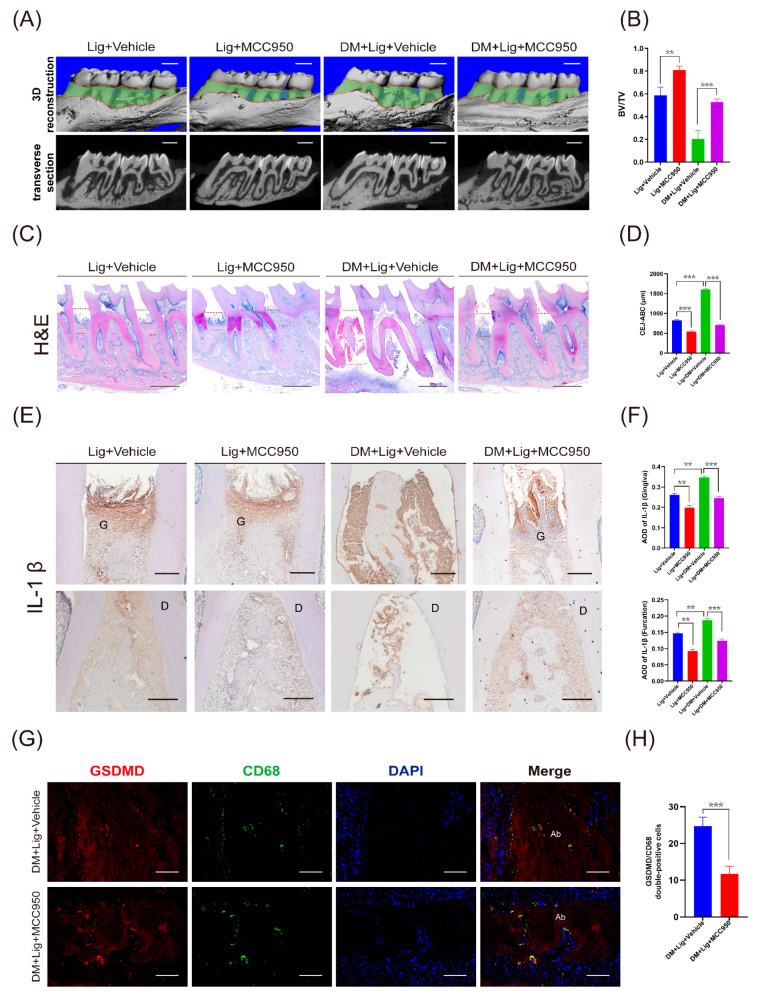
MCC950 rescued the bone loss of diabetes mellitus–periodontitis in rats. (**A**) Representative 3D reconstructions and X-ray photos of the Lig + Vehicle, Lig + MCC950, DM + Lig + Vehicle, and DM + Lig + MCC950 groups. The green area displays the exposure of the root (scale bar: 1 mm). (**B**) BV/TV of the above 4 groups was measured. (**C**) Representative images of HE staining (scale bar: 1 mm). (**D**) Distances between the CEJ and the ABC. (**E**) Immunohistochemistry staining against IL-1β of periodontal tissues (Scale bar: 200 μm). (**F**) Quantitative analysis of IL-1β in the gingiva and the furcation of the second molar. (**G**) Representative images of immunofluorescence staining of CD68 and GSDMD in the periodontium of the DM + Lig + Vehicle and DM + Lig + MCC950 groups. Nuclei were stained with DAPI (blue). Scale bar, 100 μm. (**H**) The number of GSDMD/CD68 double-stained cells was analyzed. Data in (**B**,**D**,**F**,**H**) are shown as mean ± SD (*n* = 4). ** *p* < 0.01. *** *p* < 0.001. G, gingiva; D, dentin; Ab, alveolar bone; CEJ-ABC, cementoenamel junction and alveolar bone crest; AOD, average optical density.

We then verified the effect of MCC950 on diabetes mellitus–periodontitis. MCC950 (5 μM, 20 μL) was injected locally by microsyringe in the periodontal region of the Lig + MCC950 and DM + Lig + MCC950 groups every other day. The dosage of MCC950 was based on current literature [11,17,18] and our in vitro studies. The results of micro-CT revealed that the MCC950 treatment significantly ameliorated alveolar bone loss (Figure 2A) and improved BV/TV in the Lig + MCC950 and DM + Lig + MCC950 groups compared with the Lig + Vehicle and DM + Lig + Vehicle groups, respectively (Figure 2B). HE staining also indicated that the height of the alveolar bone in the Lig + MCC950 and DM + Lig + MCC950 groups was significantly higher than the Lig + Vehicle and DM + Lig + Vehicle groups, respectively (Figure 2C,D). Moreover, immunohistochemistry staining showed that MCC950 treatment suppressed the expression of IL-1β in the Lig + MCC950 and DM + Lig + MCC950 groups (Figure 2E,F). In addition, by immunofluorescence staining, we found that MCC950 significantly decreased CD68+ and GSDMD+ cells in the DM + Lig + MCC950 group compared with the DM + Lig + Vehicle group (Figure 2G,H). These data suggested that MCC950 effectively attenuated alveolar bone resorption by suppressing macrophage pyroptosis and the expression of IL-1β when locally injected into the gingiva in experimental diabetes mellitus–periodontitis.

### 2.3. High Glucose Promotes LPS-Induced Macrophage Pyroptosis and IL-1β Secretion

**Figure 3:**
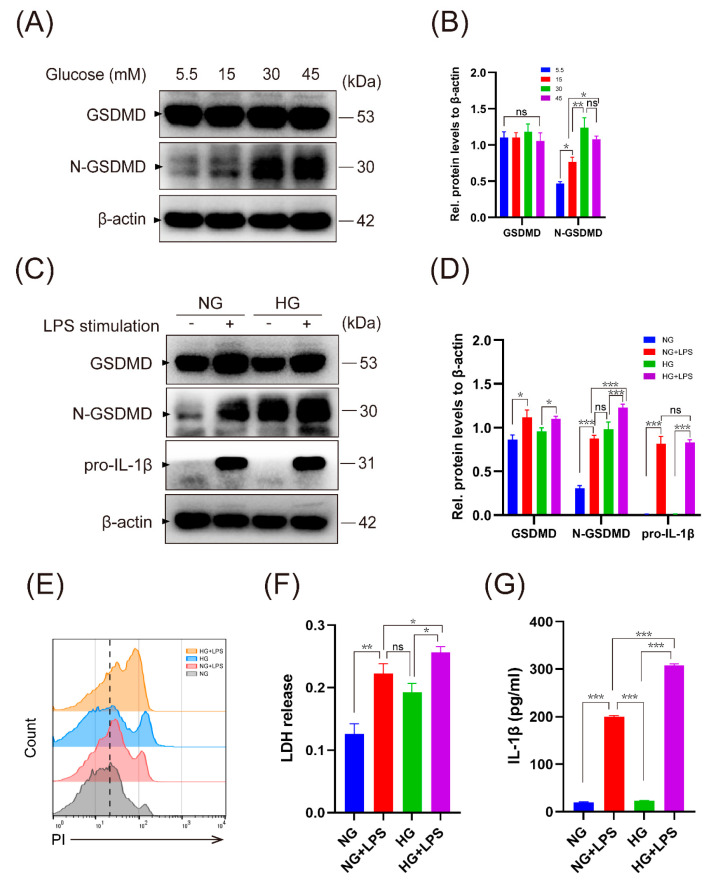
High glucose aggravated LPS-induced macrophage pyroptosis and IL-1β production. (**A**,**B**) THP-1 macrophages were treated by different concentrations of glucose for 48 h. The expression of the GSDMD and N-GSDMD proteins was assessed. (**C**–**G**) Macrophages were cultivated in normal glucose (5.5 mM, NG) or high glucose (30 mM, HG) medium for 24 h, and were then stimulated with or without LPS (1 μg/mL) for 24 h. (**C**,**D**) GSDMD, N-GSDMD and pro-IL-1β were assessed by Western blot. (**E**) Flow cytometry of macrophages stained by PI. (**F**) Analysis of LDH release to measure cell lysis. (**G**) Secretion of IL-1β in the supernatant by ELISA. Values are mean ± SD. * *p* < 0.05. ** *p* < 0.01. *** *p* < 0.001. PI, propidium iodide; LDH, lactate dehydrogenase.

To explore whether hyperglycemia played a role in the process of macrophage pyroptosis, we first incubated THP-1 macrophages under high glucose conditions. Western blot showed N-GSDMD were significantly induced when macrophages were exposed to 30 mM and 45 mM of glucose for 48 h (Figure 3A,B). However, there was little expression of pro-IL-1β, thus very low concentration of mature IL-1β in the supernatant, which was verified by ELISA (Figure S2A). These results showed that high glucose stimulation could lead to pyroptosis, but had no effect on macrophage priming.

We next treated macrophages with LPS (purified from Porphyromonas gingivalis), a pathogen-associated molecule involved in proinflammatory response. As expected, the expression of N-GSDMD was further enhanced in macrophages exposed to HG and LPS stimulation compared with LPS stimulation alone (Figure 3C,D). Pore formation was assessed by propidium iodide (PI) staining and cytolysis was assessed by the release of lactate dehydrogenase (LDH) into the supernatant. HG and LPS stimulation resulted in significantly more pore formation and cell lysis, as revealed by PI fluorescence and LDH release (Figure 3E,F). Microscopy images showed the typical morphology of pyroptotic macrophages, including swelling and membrane rupture (Figure S2B). In addition, LPS triggered the secretion of IL-1β in primed macrophages, which was further augmented in macrophages cultured in high glucose (Figure 3G and Figure S2C). However, the expression of pro-IL-1β had no significant difference between the NG + LPS group and the HG + LPS group (Figure 3C). These collective results indicated that high glucose enhanced endotoxin-induced IL-1β production at the cleavage and secretion process in macrophages.

### 2.4. High Glucose Leads to Severe Oxidative Stress Response in Macrophages Stimulated by LPS

**Figure 4:**
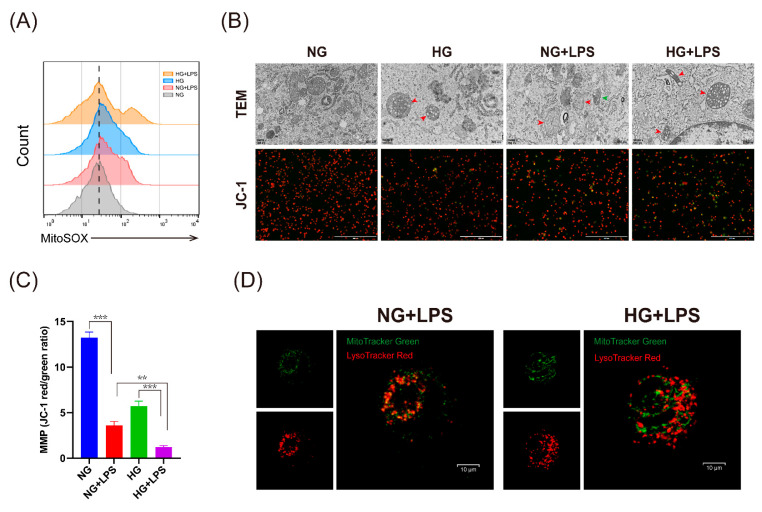
High glucose promoted ROS generation in macrophages stimulated by LPS. (**A**–**D**) THP-1 macrophages were cultivated in NG or HG medium for 24 h, and were then stimulated with or without LPS (1 μg/mL) for 24 h. (**A**) THP-1 macrophages were stained with mitoSOX and analyzed by flow cytometry. (**B**) Transmission electron microscope (TEM) images (upper panel) of THP-1 macrophages (scale bar: 500 nm). The red arrow represents dysfunctional mitochondria and the green arrow represents autolysosome. JC-1 staining (lower panel) to detect mitochondrial membrane potential (MMP) in THP-1 macrophages (scale bar: 400 μm). (**C**) Quantitative analysis of JC-1 using red/green ratio. (**D**) Macrophages were stained with MitoTracker Green and LysoTracker Red. The colocalization of mitochondria and lysosome dots were observed using confocal microscopy (scale bar: 10 μm). ** *p* < 0.01. *** *p* < 0.001. TEM, transmission electron microscope; MMP, mitochondrial membrane potential.

Previous studies suggested that ROS derived from mitochondria is essential for the activation of the NLRP3 inflammasome, which is critical to trigger an inflammatory response through the maturation of cytokines such as IL-1β [19,20]. In this study, we found that HG and LPS stimulation led to a higher level of ROS than LPS stimulation alone, as reflected by dcfh-DA (Figure S2D) and mitochondria-specific ROS indicator MitoSOX (Figure 4A). We hypothesized that the increase in mitochondrial ROS could be due to the accumulation of dysfunctional mitochondria. To test this, we used a transmission electron microscope (TEM) and JC-1 to determine the changes in mitochondria, and we observed more dysfunctional mitochondria with loss of membrane potential in macrophages stimulated by HG and LPS (Figure 4B,C). In addition, autolysosomes could be observed in macrophages of the NG + LPS group. By using MitoTracker Green and LysoTracker Red fluorescent probes, we found colocalization of mitochondria and lysosomes in macrophages stimulated by NG + LPS, which indicated the degradation of mitochondria by lysosomes (Figure 4D). The results of oxidative stress response in U937 macrophages are provided in Figure S3. These data suggested that the accumulation of dysfunctional mitochondria characterized by low membrane potential and a high level of ROS occurred during LPS stimulation in the presence of HG.

### 2.5. High Glucose Impairs Autophagy in Macrophages

**Figure 5:**
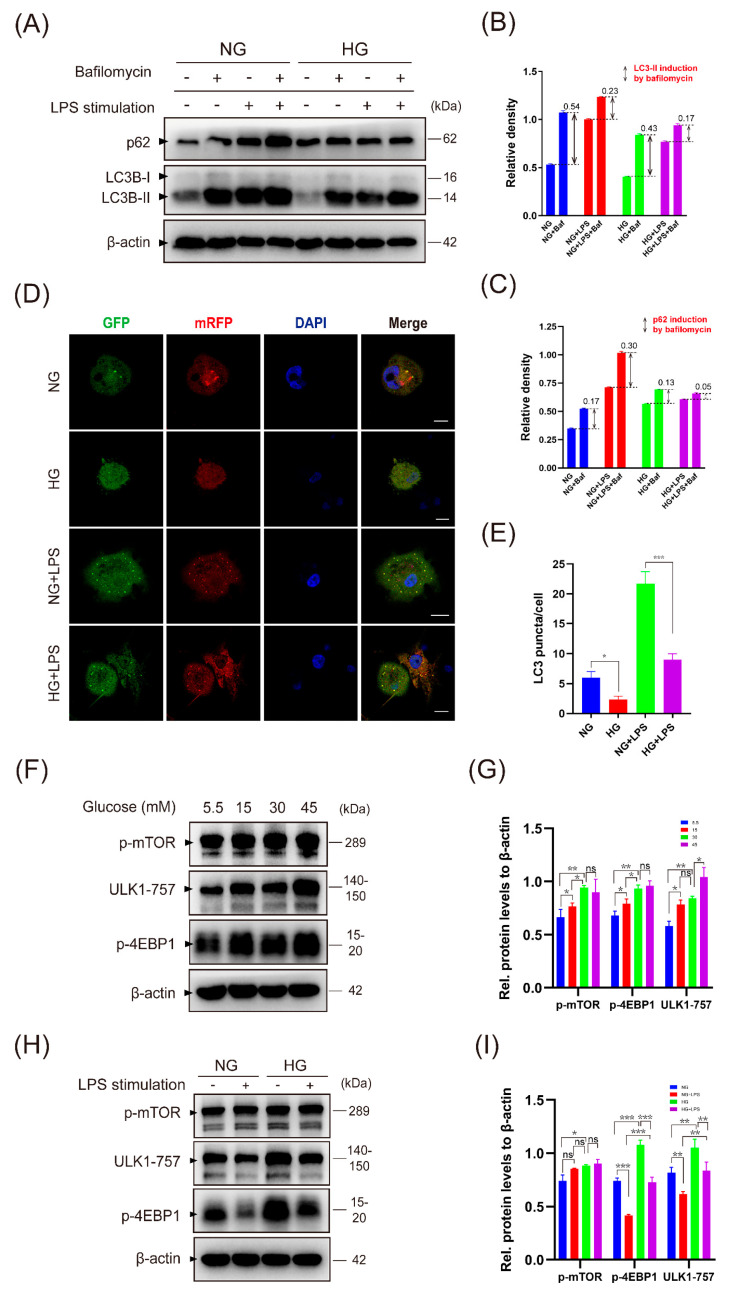
HG activates the mTOR pathway and decreases autophagic flux in THP-1 macrophages. (**A**–**C**) THP-1 macrophages were cultivated in NG or HG medium for 24 h and were then stimulated with or without LPS (1 μg/mL) for 24 h. For the last 2 h, cells were treated with or without bafilomycin (Baf, 100 nM). LC3B-II and p62 protein levels were measured by Western blot and quantified using ImageJ analysis. LC3B-II and p62 induction by bafilomycin demonstrates autophagic flux. (**D**,**E**) Macrophages were transfected with mRFP-GFP-LC3 adenovirus for 6 h (MOI = 200) and were then stimulated as illustrated above. (**D**) Representative microscopic images of LC3 puncta (scale bar: 10 μm). (**E**) Quantification based on counting LC3 puncta per cell. (**F**–**I**) Detection of mTOR pathway expression of THP-1 macrophages stimulated as in Figure 3A (**F**,**G**) and Figure 3C (**H**,**I**). Values are mean ± SD. * *p* < 0.05. ** *p* < 0.01. *** *p* < 0.001.

We proposed that the accumulation of ROS-generating mitochondria was due to defective autophagy. Western blot showed that LC3B and p62 levels increased significantly in macrophages during LPS stimulation. However, in the presence of HG, autophagy activity was inhibited as reflected by the reduction in LC3B. To determine the changes in autophagic flux, we used bafilomycin to prevent the fusion of autophagosomes and lysosomes. Consistent with the inhibition of autophagosome degradation, bafilomycin resulted in LC3B and p62 accumulation. The results of the LC3B and p62 induction by bafilomycin demonstrate a decrease in autophagic flux with HG stimulation, no matter with or without LPS stimulation (Figure 5A–C). These results were further confirmed by measuring LC3 puncta via transfection of adenovirus as illustrated in Figure 5D,E, which suggested impaired autophagy activity in macrophages under high-glucose culture.

mTOR is a key metabolic regulator, and the activation of mTORC1 controls glucose metabolism and inhibits autophagy [21]. Stimulation of THP-1 macrophages with high glucose resulted in mTORC1 activation, as indicated by increased phosphorylation of mTOR and the downstream target such as 4E-BP1, which reached the maximal level in 45 mM glucose (Figure 5F,G). High glucose also prevents ULK1 (a central kinase involved in autophagy initiation) activation by phosphorylating ULK1 at Ser 757 (Figure 5F). The expression of 4E-BP1 and ULK1-757 was reduced by LPS stimulation but still remained at a high level in macrophages stimulated by HG and LPS, which was consistent with the results that high glucose suppressed the autophagic flux (Figure 5H,I). The results of autophagy and the mTOR pathway in a rat model (Figure S1C,D) and U937 macrophages (Figure S4) are provided in the Supplementary Materials.

### 2.6. Down-Regulated ROS-Inflammasome Pathway Alleviates Macrophage Pyroptosis

**Figure 6:**
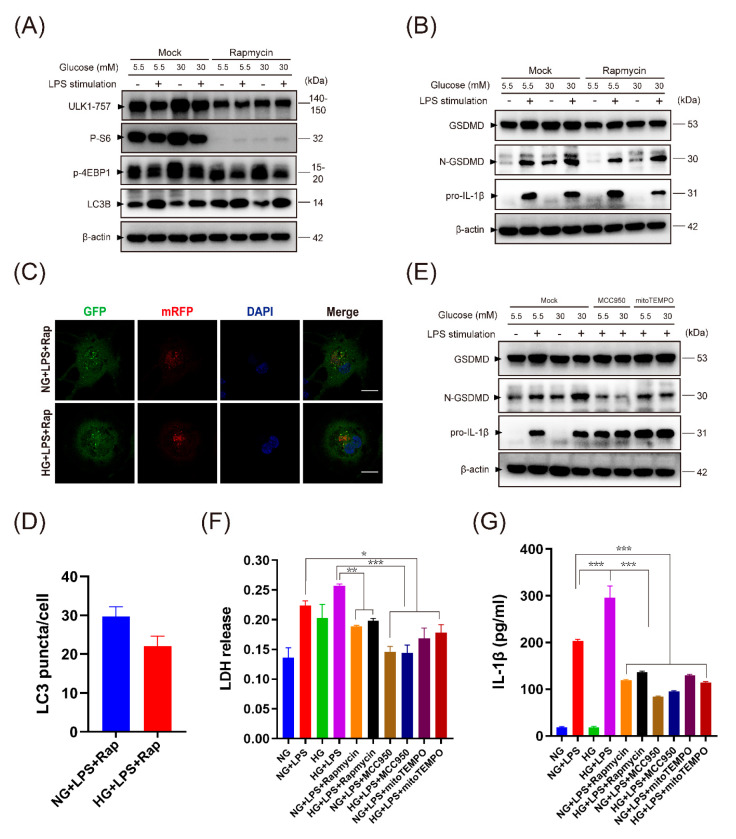
Modulation of autophagy and ROS-inflammasome pathway reduced IL-1β secretion and pyroptosis. (**A**–**D**) Macrophages were pretreated with rapamycin (200 nM) 2 h before LPS stimulation. Proteins related to mTOR pathway, autophagy (**A**), and pyroptosis (**B**) were assessed by Western blot. (**C**,**D**) Representative microscopic images and quantification of LC3 puncta (scale bar: 10 μm). (**E**) MCC950 (5 μM) and mitoTEMPO (10 μM) were added to the media 2 h before LPS stimulation and were maintained until assays were performed. GSDMD, N-GSDMD, and pro-IL-1β were measured. (**F**) LDH in the supernatant to measure cell lysis. (**G**) Measurement of IL-1β by ELISA. Data in (**D**,**F**,**G**) are shown as mean ± SD. * *p* < 0.05. ** *p* < 0.01. *** *p* < 0.001. LDH, lactate dehydrogenase.

We next treated macrophages with rapamycin to directly inhibit the mTOR pathway and downregulate ser-ulk1-757 (Figure 6A). Rapamycin could effectively activate autophagy as revealed by increased expression of LC3B (Figure 6A,C,D) and reduce accumulation of dysfunctional mitochondria with loss of membrane potential and ROS generation during HG and LPS stimulation (Figure S5A). In addition, rapamycin treatment could also reduce N-GSDMD expression, LDH release, and IL-1β secretion in macrophages stimulated by LPS (Figure 6B,F,G). To test whether mitochondrial ROS was the trigger of the inflammasome and pyroptosis, we pretreated THP-1 macrophages with MitoTEMPO (10 μM) 2 h before LPS stimulation. MitoTEMPO could reduce mitochondrial ROS generation (Figure S5B), macrophage pyroptosis, and IL-1β secretion (Figure 6E–G). In addition, we treated THP-1 macrophages with MCC950 (5 μM), a small molecule inhibitor of the NLRP3 inflammasome. Immunoblot of N-GSDMD, LDH release, and ELISA revealed that MCC950 could effectively salvage pyroptosis and reduce IL-1β secretion (Figure 6E–G). Quantification of Figure 6A,B,E was provided in Figure S6.

## 3. Discussion

**Figure 7:**
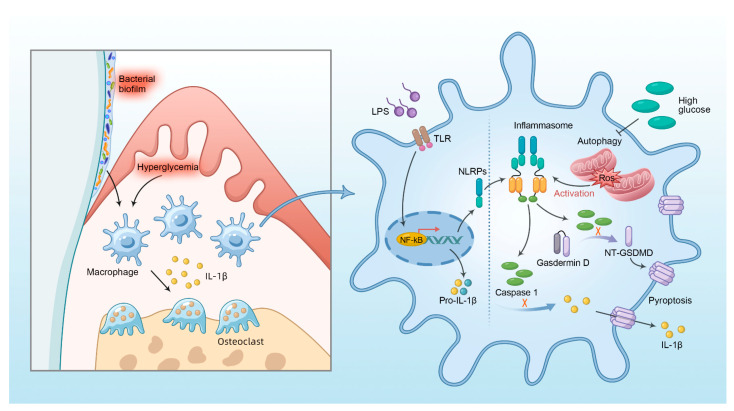
Diagram illustrates that hyperglycemia could promote macrophage pyroptosis via autophagy impairment and ROS-inflammasome pathway and aggravate inflammatory diseases such as periodontitis.

In this study, we investigated the impact of macrophage pyroptosis on the development of diabetes mellitus–periodontitis. Our results revealed the following aspects in diabetes mellitus–periodontitis: (1) hyperglycemia aggravates periodontitis by promoting macrophage pyroptosis and the secretion of inflammatory cytokine IL-1β and (2) high glucose impairs autophagy via mTOR-ULK1 pathway, leading to the accumulation of ROS-generating mitochondria, and this in turn promotes inflammasome activation and pyroptosis in macrophages stimulated by LPS (Figure 7). This study will improve understanding of the molecular mechanisms involved in the pathology of diabetes–periodontitis, which may provide new insights into novel strategies or targets to improve diabetes mellitus–periodontitis.

Multiple studies indicate that diabetes mellitus is an established risk factor for periodontitis [1,22,23]. Diabetes mellitus changes the microenvironment of periodontal tissues, including complex periodontal plaque, oxidative stress and immunosuppression, which leads to a hyperinflammatory response to the periodontal microbiota. In this study, we observed the expression of GSDMD in the gingiva of diabetic rats, in agreement with a recent study, which showed GSDMD+ cells were increased in the gingival tissue of diabetic mice [10]. In addition, aberrant macrophage pyroptosis and expression of IL-1β could be observed in periodontal lesions of rats with diabetes mellitus–periodontitis. Furthermore, we found that high glucose promoted secretion of LPS-primed IL-1β via activation of inflammasome and pore formation at the plasma membrane in THP-1 macrophages. This is consistent with a previous clinical study which revealed that diabetics had significantly higher levels of IL-1β in GCF compared with non-diabetic controls who were matched in periodontal disease severity [24]. These studies demonstrate that diabetes mellitus may interact synergistically with Gram-negative bacteria to yield high levels of inflammatory mediators and aggravate periodontal condition.

IL-1β acts as a strong stimulator of bone resorption and has been identified as a salivary biomarker for discriminating periodontitis in type II diabetes mellitus [25]. This is also confirmed by our observation of high expression of IL-1β and aggravated destruction of periodontal tissues in rats with diabetes mellitus–periodontitis. The hyperinflammatory response and destruction could be significantly alleviated by treatment of MCC950 locally in the periodontal region. MCC950 is a selective molecule inhibitor of the NLRP3 inflammasome. Coll et al. demonstrate that MCC950 does not block the major anti-microbial inflammasomes NLRC4 and NLRP1, which means it will not completely block IL-1β and antimicrobial responses may remain intact during infection [11]. The effects of MCC950 on inhibiting inflammasome activation have been tested in various models [13,16,17,26]. In this study, we found that MCC950 reduced macrophage pyroptosis, suppressed IL-1β secretion, and ameliorated alveolar bone loss, suggesting that targeting inflammasome-mediated inflammation is a novel therapeutic strategy for the treatment of diabetes–periodontitis.

Activation of inflammasome is critical for cleavage of pro-IL-1β and GSDMD. A previous study suggested that NLRP3 inflammasome is negatively regulated by autophagy and positively regulated by ROS derived from damaged mitochondria [20]. As in our study, we found that high glucose and LPS stimulation led to the accumulation of dysfunctional mitochondria and robust generation of ROS. The results are coordinated to a previous study, which revealed that significantly severer mitochondrial oxidative stress and dysfunction were found in rats with diabetic periodontitis than the others [27]. Scavenging mitochondrial ROS by mitoTEMPO effectively reduced pyroptosis and IL-1β secretion, which indicated that ROS is critical for the activation of inflammasome and pyroptosis. Interestingly, Evavold et al. demonstrate that the Ragulator-Rag-mTORC1 pathway promotes ROS production, which promotes GSDMD oligomerization and pore formation, but not the cleavage of GSDMD [28]. In our study, the expression of N-GSDMD significantly increased after LPS and high glucose stimulation, so more work needs to be conducted to verify whether ROS promotes the activation of inflammasome and cleavage of GSDMD or the oligomerization of GSDMD in the plasma of macrophages.

Previous studies demonstrate that the deletion of autophagic proteins enhances inflammasome activation and secretion of IL-1β stimulated by LPS in macrophages, which requires ROS generation [29,30]. Here we show that, upon LPS activation, THP-1 macrophages cultured in high glucose had further reduced autophagic flux compared with macrophages in normal glucose, as reflected by LC3B and p62 induction by bafilomycin. These results are consistent with previous studies that high glucose suppresses autophagy in various cells [31,32,33]. LPS stimulation can lead to mitochondria damage and the generation of ROS [34]. However, as dysfunctional mitochondria were eliminated by lysosomes in the process of autophagy, oxidative stress was at a low level. When cultured in high glucose, macrophages were subjected to severe oxidative stress as a result of impairment of autophagy and accumulation of damaged mitochondria.

mTOR activity reflects cellular nutritional status and the inhibitory function of mTOR in autophagy is well established [35,36,37]. Here, we show that high glucose promotes mTOR activation and the phosphorylation of ULK1 at Ser 757. This is consistent with a previous study, which demonstrates that under nutrient sufficiency, high mTOR activity prevents ULK1 activation by phosphorylating ULK1 at Ser 757 and disrupting its interaction with AMPK [38]. In another study, phosphorylation of ULK1 at Ser 555 by AMPK is required for ULK1 function and mitophagy in the response to nutrient deprivation [39]. ULK1 activation is critical for the induction of autophagy and regulation of mitophagy [40,41,42]. When we treated THP-1 macrophages with rapamycin to downregulate the mTOR pathway, rapamycin significantly increased autophagic flux, saved mitochondria fitness, and reduced ROS generation and pyroptosis.

Several limitations of our study need to be pointed out. First, in the in vivo study, we examined proteins involved in autophagy, such as LC3B and ULK1-757. More work needs to be conducted to verify autophagy activity in periodontal tissues, such as observation of autophagosome and mitochondria by TEM. Second, the in vitro setting should be modified, such as by using primary macrophages in the periodontal tissues. Third, we used MCC950 to indirectly proof NLRP3 inflammasome-mediated pyroptosis and we will further explore the expression of caspase-1 and other inflammasomes involved in macrophage pyroptosis.

## 4. Materials and Methods

### 4.1. Animal Model

All animal procedures were performed in accordance with the guidelines of Chongqing Medical University Institutional Animal Care and Use Committee (Ethics Number: 2022-092) and the ARRIVE guidelines. Sprague–Dawley rats (male, 4-week-old, 100 ± 8 g) were purchased from Chongqing Medical University. Rats were randomly divided into 4 groups, including control (CON), diabetes mellitus (DM), ligature-induced periodontitis (Lig), and DM + Lig groups (*n* = 8/group). The sample size was calculated by Power Analysis and Sample Size software (version 15.0). To establish the type 2 diabetes model, rats were fed high-fat and high-sugar foods (Boaigang, Beijing, China) for 4 weeks. After 8 h of fasting, rats received intraperitoneal injection of streptozotocin (40 mg/kg, S0130, Sigma, Darmstadt, Germany). Rats in the control and Lig groups were injected with the same volume of citrate buffer (C1010, Solarbio, Beijing, China). A total of 1 week after injection of STZ, fasting blood glucose (FBG) was tested and rats with FBG ≥ 16.6 mmol/L were considered diabetic. About 2 weeks after injection of STZ, we placed 3-0 silk sutures around the maxillary second molars after anesthesia to induce periodontitis. Ligatures were checked every other day and loose ligatures were placed back in time. Four weeks after the induction of periodontitis, rats were euthanized for further analysis. For the intervention experiment, rats were randomly divided into four groups, including Lig + Vehicle, Lig + MCC950, DM + Lig + Vehicle, and DM + Lig + MCC950 groups (*n* = 8/group). For treatment, MCC950 (5 μM, 20 μL, HY-12815, MedChemExpress, Monmouth Junction, NJ, USA) was injected with a microsyringe (Hamilton, Switzerland) in the periodontal region once every 2 days from the day the ligatures were installed. Rats were euthanized for further analysis four weeks later. The dosage of MCC950 was based on current literature [11,17,18] and our in vitro studies.

### 4.2. Cell Culture and Stimulation

THP-1 cells and U937 cells from passage 3 to passage 8 were used in this study. THP-1 cells and U937 cells were obtained from the Cell Bank of the Chinese Academy of Sciences (Shanghai, China) and incubated in 1640 RPMI media (PM150110, Procell, Wuhan, China) supplemented with 10% FBS (Gemini, Santa Ana, CA, USA) and 1% penicillin–streptomycin (15070063, Invitrogen, Carlsbad, CA, USA). Cells were induced to differentiate into adherent macrophages by incubation in the presence of 100 ng/mL phorbol 12-myristate 13-acetate (P1585, PMA, Sigma, Darmstadt, Germany) for 3 days. Cells were then washed with phosphate-buffered saline and the attached macrophages were incubated with the complete culture media without PMA for 24 h before the experiment.

For high-glucose stimulation only, macrophages were cultured in media with different concentrations of glucose (5.5 mM, 15 mM, 30 mM, 40 mM) by adding D-glucose (G8150, Solarbio, Beijing, China) to sugar-free 1640 RPMI culture medium (PM150122, Procell, Wuhan, China) for 48 h [31,43]. For high-glucose and LPS stimulation, macrophages were first incubated with the complete culture media containing 5.5 mM glucose (normal glucose condition) or 30 mM glucose (high-glucose condition) for 24 h and then stimulated in the presence of 1 mg/mL LPS (SMB00610, Sigma, Darmstadt, Germany) for 24 h. For treatment, rapamycin (200 nM, V900930, Sigma, Darmstadt, Germany) [43,44], MCC950 (5 μM, HY-12815, MedChemExpress, NJ, USA) [11,18], and mitoTEMPO (10 μM, HY-112879, MedChemExpress, NJ, USA) [45,46] were added to the media 2 h before LPS stimulation.

### 4.3. Micro-Computed Tomography

After fixation in 4% formaldehyde for 48 h, maxillary alveolar bones were scanned with a micro-computed tomography system (μCT40, SCANCO, Wangen-Brüttisellen, Switzerland) with a voxel resolution of 10 μm. Three dimensional reconstructions and X-ray images were performed to indicate the absorption of alveolar bones. Bone volume to tissue volume ratio (BV/TV) was measured by Scanco Medical analysis software (Version 6.1). The 200 μm × 200 μm rectangular area of the alveolar bone under the furcation of the maxillary second molar was defined as the region of interest (ROI).

### 4.4. Histological Staining

Maxillae were decalcified in 20% ethylenediaminetetraacetic acid for 2 months and embedded in paraffin. Hematoxylin and eosin staining, trap staining, and immunohistochemistry and immunofluorescence staining were processed. Maxillae were fixed in 4% formaldehyde (BL539A, Biosharp, Guangzhou, China) for 48 h and were decalcified in 20% ethylenediaminetetraacetic acid (V900081, Sigma, Darmstadt, Germany) for 2 months. The specimens were embedded in paraffin (Leica, Wetzlar, Germany) and were cut into 5 μm sections for histologic analysis. Hematoxylin and eosin staining was processed by using the HE Stain Kit (G1120, Solarbio, Beijing, China) and trap staining was processed by using the Tartrate-Resistant Acid Phosphatase (TRAP) Stain Kit (G1050-50T, Servicebio, Wuhan, China) according to instructions. Under the furcation of the second molar, a fixed size of vision was selected to calculate the number of osteoclasts in the alveolar bone. Three tissue sections were calculated in each group. For immunohistochemistry staining, sections were rehydrated with graded ethanol and incubated with 3% hydrogen peroxide for 15 min at room temperature to block the activity of endogenous peroxidase. Then, the sections were incubated with antigen retrieval solution for 15 min at 95 °C, blocked by 5% goat serum (ZLI-9022, ZSG-Bio, Beijing, China) for 30 min and incubated with primary antibodies against cleaved-IL-1β (#AF4006, 1:300, Affinity Biosciences, Cincinnati, OH, USA), GSDMD (#39754, 1:200, Cell Signaling Technology, Danvers, MA, USA) or Phospho-ULK1 (Ser757) (1:200, #AF4387, Affinity Biosciences, USA) overnight. The sections of negative control were incubated with dilution buffer without primary antibodies. Appropriate secondary antibodies (PV-9001, ZSGB-Bio, Beijing, China) were applied subsequently. After the production of brown precipitation with DAB detection kit (ZLI-9017, ZSGB-Bio, Beijing, China), sections were counterstained with hematoxylin. AOD (average optical density) was measured by ImageJ software (Version 1.54b) to quantify the expression of cleaved-IL-1β and GSDMD (total GSDMD and cleaved GSDMD). Images of negative control of IL-1β, GSDMD and ULK1-757 was provided in Figure S7. For immunofluorescence staining, sections were incubated with primary antibodies against GSDMD (1:200, #39754, Cell Signaling Technology, Danvers, MA, USA) or CD68 (1:200, ab283654, Abcam, Cambridge, UK) overnight, and then incubated with fluorescent secondary antibodies (ab150077, ab150078, Abcam, Cambridge, UK). Sections were counterstained by DAPI for 5 min and then observed under a fluorescence microscope (ZEISS, Oberkochen, Germany). Co-localization of GSDMD and CD68 was analyzed by ImageJ. For immunohistochemistry/immunofluorescence staining, we chose three samples from each experimental group and three tissue slices from each sample for staining and analysis.

### 4.5. Western Blot Analysis

Macrophages stimulated as described above were lysed by lysis buffer. The concentration of protein was determined by the BCA Protein Assay Kit (ZJ102, Epizyme Biotech, Shanghai, China). Appropriate gels (7.5%, 10%, 12.5%, PG211, PG212, PG213, Epizyme Biotech, Shanghai, China) were used in this experiment. After electrophoresis, proteins were transferred to PVDF membranes (Millipore, Burlington, MA, USA) and incubated in 5% skim milk at room temperature for 1 h. PVDF membranes were then incubated in primary antibody against GSDMD (#39754), N-GSDMD (#39754), pro-IL-1β (#12242), *p*-mTOR (#5536), phospho-ULK1 (Ser757) (#14202), p-S6 (#4858), p-4EBP1 (#2855), mTOR (#2983), Raptor (#2280), LC3B (#58139), p62 (#5114), and β-actin (#4970) (1:1000, all from Cell Signaling Technology, Danvers, MA, USA) at 4 °C overnight. After being washed with TBST 3 times, membranes were incubated with corresponding secondary antibodies (1:5000, ab6721, Abcam, Cambridge, UK) for 2 h. The ECL kit (SQ201, Epizyme Biotech, Shanghai, China) was used to detect the protein in the membrane and ImageJ software (Version 1.54b) was used for protein quantification. Three replicates were used in Western blot.

### 4.6. PI Staining

THP-1 macrophages grown in 60 mm plastic culture dishes were stimulated as described above. Cells were stained with diluted PI solution (3.33 μg/mL, P4864, Sigma, Darmstadt, Germany) for 20 min at 37 °C in the dark. For flow cytometry, data were acquired with a flow cytometer (BD Biosciences, Franklin Lakes, NJ, USA) and analyzed with FlowJo software (version 10.6.2).

### 4.7. LDH Release Assay

1E5 cells per well were seeded into a 96-well plate and stimulated as described above. The plate was spun at 400× *g* for 5 min to ensure all cells were at the bottom of the well. Supernatants from the plate were collected to detect LDH release by the LDH Cytotoxicity Assay Kit (C0016, Beyotime, Shanghai, China) per the manufacturer’s instructions.

### 4.8. Enzyme-Linked Immunosorbent Assay

IL-1β release in supernatants was measured by the Human IL-1β ELISA Kit (EK101B, MULTISCIENCES, Hangzhou, China) according to the manufacturer’s protocols.

### 4.9. Measurement of Mitochondrial Contents

THP-1 macrophages seeded in culture plates were stimulated as described above. Dcfh-DA (for ROS, 35845, Sigma, Darmstadt, Germany), MitoSOX (for mitochondrial ROS, M36008, Invitrogen, CA, USA), JC-1 (for mitochondrial membrane potential, C2003S, Beyotime, Shanghai, China), MitoTracker Green (for total mitochondria, M7514, Invitrogen, CA, USA), and LysoTracker Red (for lysosomes, L7528, Invitrogen, CA, USA) staining were performed according to the manufacturer’s instructions. For dcfh-DA and JC-1 staining, cells were observed by fluorescence microscope AE31 (Olympus, Tokyo, Japan). MitoSOX staining was analyzed by flow cytometry. For MitoTracker Green and LysoTracker Red staining, cells were observed by Leica AF6000 Modular System (Leica, Germany).

### 4.10. Electron Microscopy

Cultured cells were centrifuged at 1200 rpm for 10 min. The cell mass at the bottom was fixed with 2.5% glutaraldehyde in 0.1 M sodium cacodylate buffer and was successively put into ethanol for dehydration. Ultrathin sections of 50 nm were made after polymerization with uranyl acetate and lead citrate staining. The morphology of mitochondria was observed by an electron microscope (JEOL JEM-1400Plus, Tokyo, Japan).

### 4.11. mRFP-GFP-LC3 Adenoviral Vectors

Macrophages were transfected with mRFP-GFP-LC3 adenoviral vectors (HANBIO, Shanghai, China) according to the manufacturer’s instructions. LC3 puncta were examined with confocal microscope (Leica, Germany).

### 4.12. Statistical Analysis

Data analysis and charts were performed with GraphPad Prism 8.0 (GraphPad Software, La Jolla, CA, USA). All numerical results are presented as means ± standard deviations. Data normality and homogeneity were checked before proceeding to one-way ANOVA. The data were analyzed by one-way analysis of variance (ANOVA) with Tukey’s post hoc test for multiple comparisons. *p* < 0.05 was considered statistically significant (* *p* < 0.05, ** *p* < 0.01, and *** *p* < 0.001).

## 5. Conclusions

Taken together, our data reveal a role for hyperglycemia in promoting the development of periodontitis via suppression of autophagy and aberrant activation of ROS-inflammasome-mediated pyroptosis in macrophages. Defects in autophagy can result in severe oxidative stress, which leads to hyperinflammatory response, as seen in THP-1 macrophages stimulated with high glucose and LPS. Therefore, modulation of autophagy activity, clearance of ROS, and intervention of inflammasome could be beneficial for treatment or prevention of diabetes–periodontitis.

## Data Availability

The data that support the findings of this study are available from the corresponding author upon reasonable request.

## Supplementary Materials

The supporting information can be downloaded at: https://www.mdpi.com/article/10.3390/ijms24076309/s1.

## Abbreviations

LPS	Lipopolysaccharide
DM	Diabetes mellitus
STZ	Streptozocin
CEJ-ABC	Cementoenamel junction and alveolar bone crest
ELISA	Enzyme-linked immunosorbent assay
PI	Propidium iodide
LDH	Lactate dehydrogenase
ROS	Reactive oxygen species
TEM	Transmission electron microscope
MMP	Mitochondrial membrane potential
FBG	Fasting blood glucose
PMA	Phorbol 12-myristate 13-acetate
ROI	Region of interest
TRAP	Tartrate-resistant acid phosphatase
AOD	Average optical density
