# Supplementary material for: Hyperglycemia Aggravates Periodontitis via Autophagy Impairment and ROS-Inflammasome-Mediated Macrophage Pyroptosis
Source: Int J Mol Sci. 2023 Mar 27;24(7):6309. doi: 10.3390/ijms24076309 (PMC10094233; doi:10.3390/ijms24076309)
Supplement: Supplementary file 1 [file ijms-24-06309-s001.zip › ijms-2233731-supplementary.pdf]

Hyperglycemia aggravates periodontitis via autophagy impairment and ROS-inflammasome-mediated macrophage pyroptosis

Zhenxing Zhao[1,2,3] | Ye Ming[1,2,3] | Xiang Li[1,2,3] | Hao Tan[1,2,3] | Xinyi He[1,2,3] | Lan Yang[1,2,3] | Jinlin Song[1,2,3] | Leilei Zheng[1,2,3]

[1]College of Stomatology, Chongqing Medical University, Chongqing, China
[2]Chongqing Key Laboratory of Oral Diseases and Biomedical Sciences, Chongqing, China
[3]Chongqing Municipal Key Laboratory of Oral Biomedical Engineering of Higher Education, Chongqing, China

Correspondence:

Prof. Leilei Zheng
College of Stomatology, Chongqing Medical University, Songshi North Road, #426, Yubei District, Chongqing 401147, China.
E-mail: zhengleileicqmu@hospital.cqmu.edu.cn

Prof. Jinlin Song
College of Stomatology, Chongqing Medical University, Songshi North Road, #426, Yubei District, Chongqing 401147, China.
E-mail: songjinlin@hospital.cqmu.edu.cn

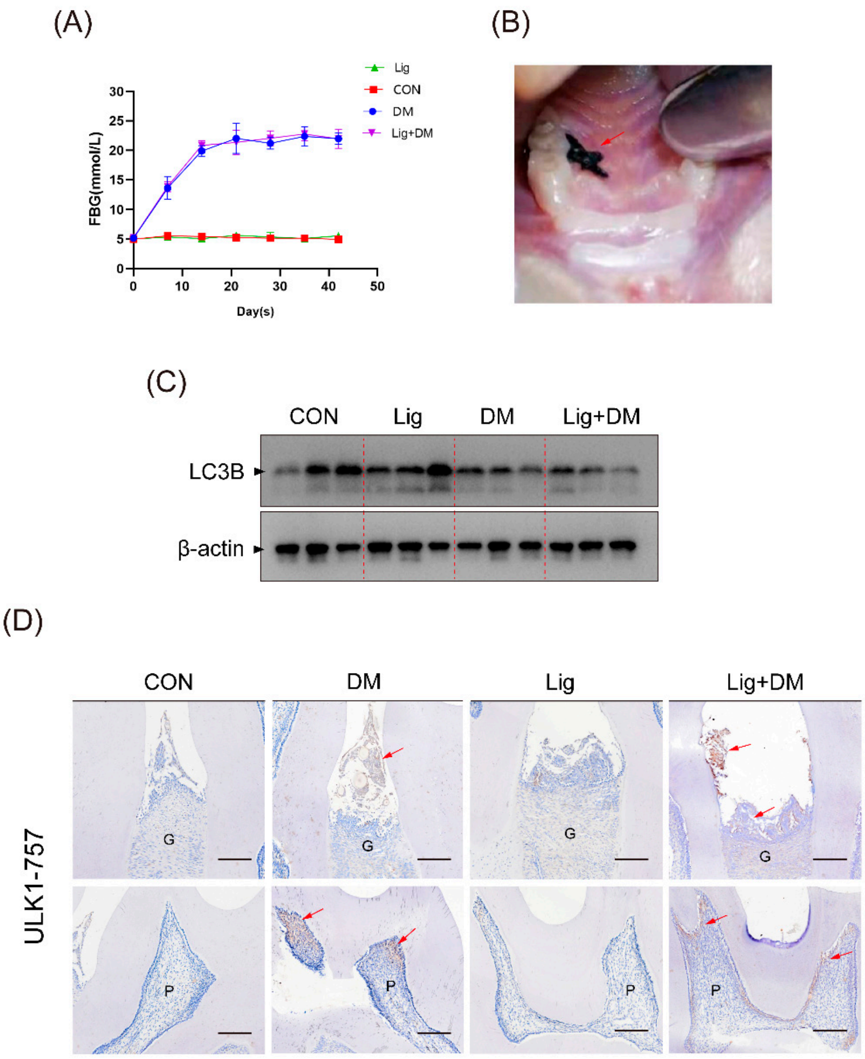

Figure S1. (A) Fasting blood glucose was tested once a week after injection of streptozotocin (STZ). Tests were performed until rats were euthanized for further analysis. (B) 3-0 silk suture was inserted around maxillary second molar as imaged. (C) Gingiva around second molar were extracted, grinded and then lysed by lysis buffer. The results of western blot reveled that the expression of LC3B decreased in DM group and DM+Lig group. (D) Immunohistochemistry staining against ULK1-757 (scale bar: 200 μm). Rats in DM group and DM+Lig group had more ULK1-757 positive cells (gingiva and pulp) than CON group and Lig group. Red arrows demonstrate ULK1-757 positive cells. G gingiva, P pulp.

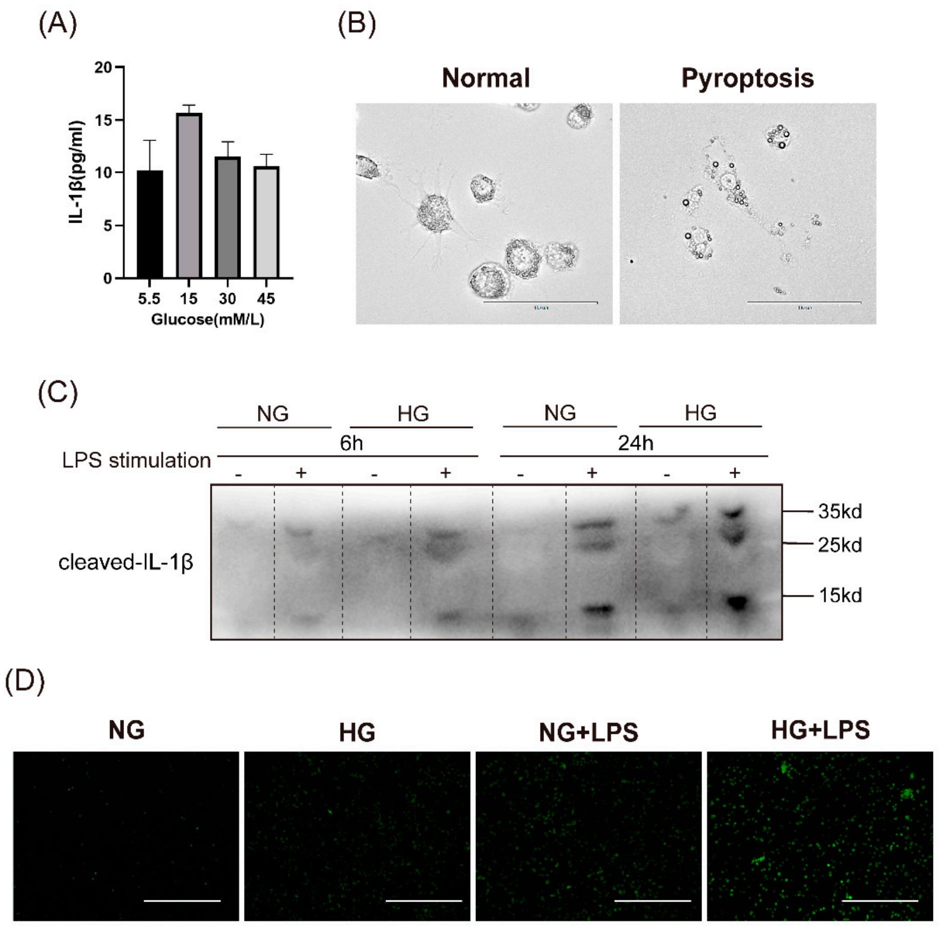

Figure S2. (A) THP-1 macrophages were treated by different concentration of glucose for 48h. IL-1β in the supernatant was measured by ELISA. (B) Typical morphology of normal and pyroptotic THP-1 macrophages (scale bar: 100 μm). (C) Macrophages were cultivated in normal glucose or high glucose medium for 24h, and then were stimulated with or without LPS (1μg/ml) for 6h or 24h. Cell supernatants were clarified by brief centrifugation and were concentrated using 10-kDa nominal molecular mass cutoff filters (Millipore, Germany). A total of 15 ug protein were resolved in 4-20% polyacrylamide gels, transferred to PVDF membranes and immunoblotted with IL-1β (1:1000, Cell Signaling Technology, Danvers, MA, USA). (D) THP-1 macrophages stained by dcfh-DA (scale bar: 1000 μm).

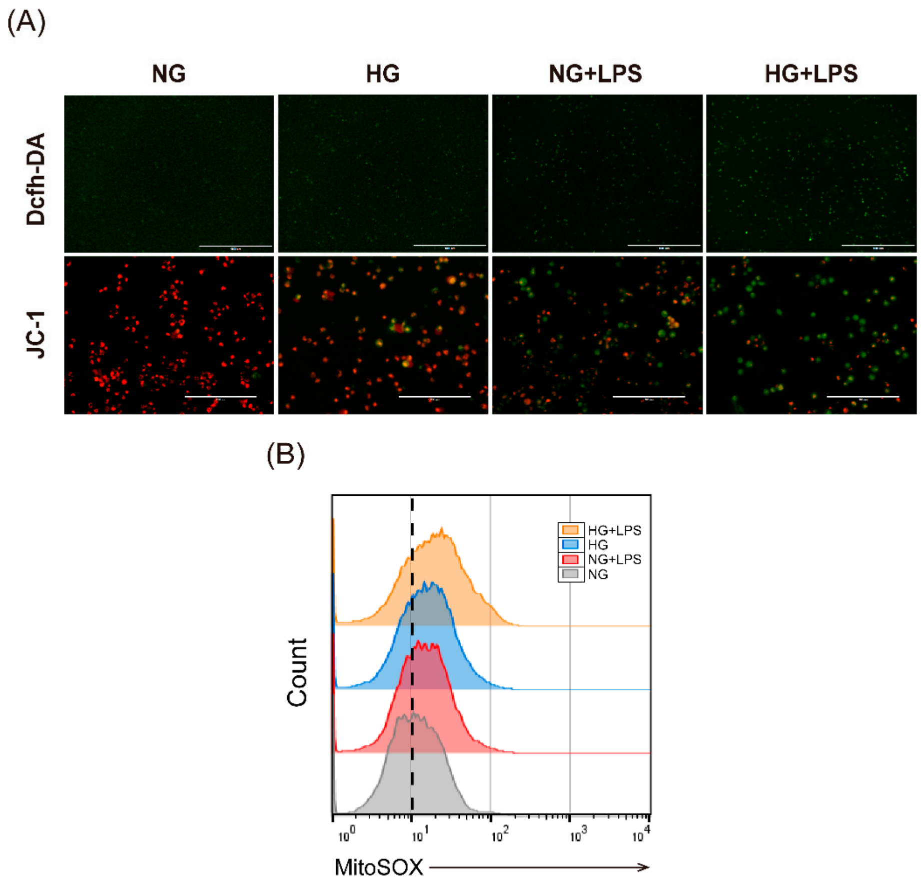

Figure S3. U937 macrophages were incubated in normal glucose or high glucose medium for 24h and then stimulated by LPS (1 mg/mL) for 24 hours. (A) ROS and mitochondrial membrane potential (MMP) were analyzed in cells labeled with dcfh-DA (scale bar: 1000 μm) or with JC-1 (scale bar: 200 μm). (B) Macrophages were stained with mitoSOX and analysed by flow cytometry to detect mitochondrial ROS.

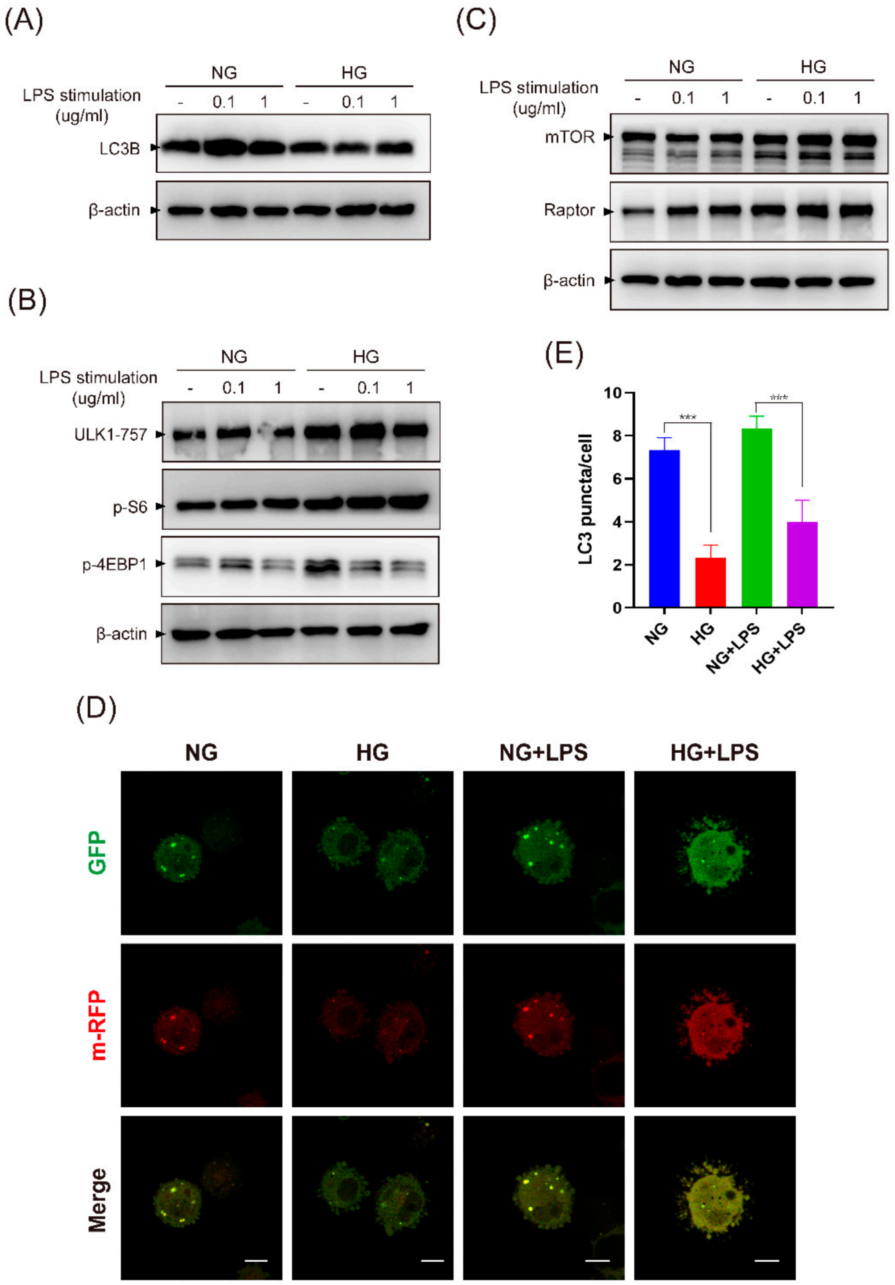

Figure S4. (A-C) U937 macrophages were incubated in normal glucose or high glucose medium for 24h and then stimulated by LPS (0.1mg/ml, 1 mg/mL) for 24 hours. Proteins related to autophagy (A) and mTOR pathway (B-C) were assessed by western blot. (D-E) U937 macrophages were transfected with mRFP-GFP-LC3 adenovirus for 6h (MOI=200). (D) Representative microscopic images of LC3 puncta (scale bar: 10 μm). (E) Quantification based on counting LC3 puncta per cell. Values are mean ± SD. ***$p < 0.001$.

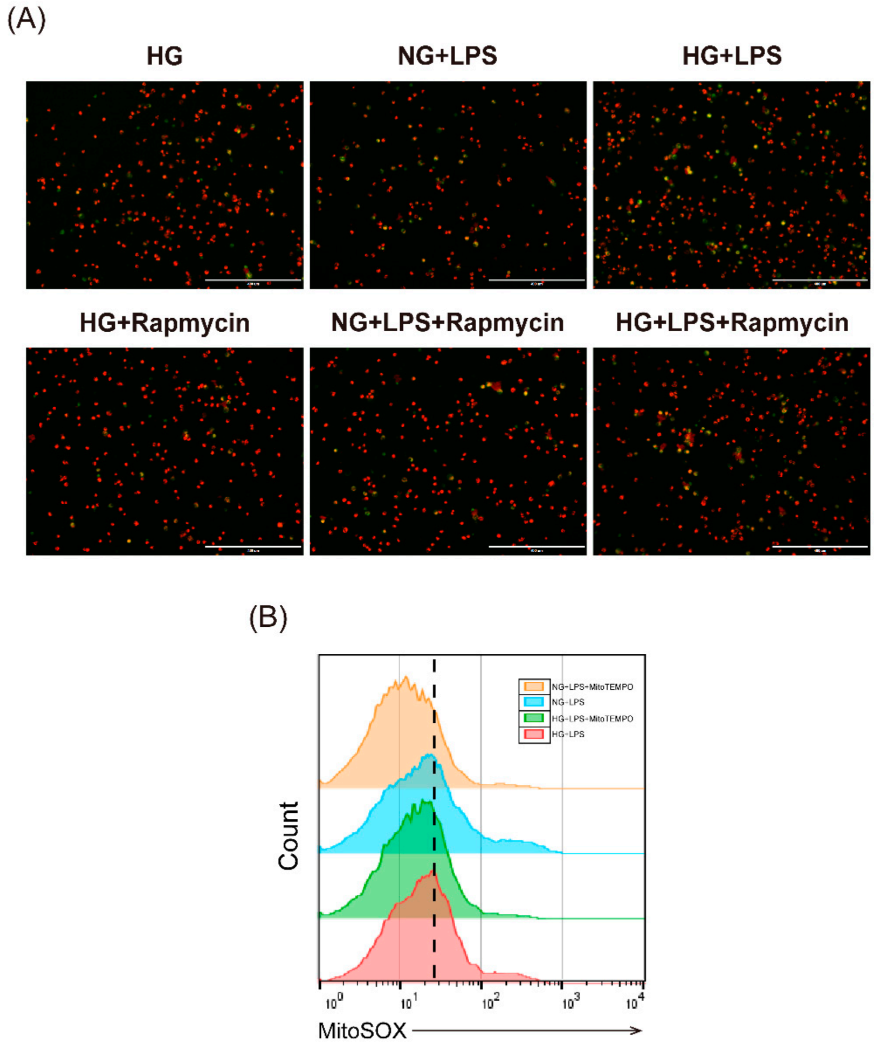

Figure S5. (A) THP-1 macrophages were cultivated in NG or HG medium for 24h. Macrophages were pretreated with rapamycin (200 nM) 2 h before LPS stimulation. JC-1 staining to detect mitochondrial membrane potential in THP-1 macrophages (scale bar: 400 μm). (B) mitoTEMPO (10 μM) were added to the media 2 h before LPS stimulation and were maintained until assays were performed. MitoSOX staining was performed to detect ROS generated from mitochondria.

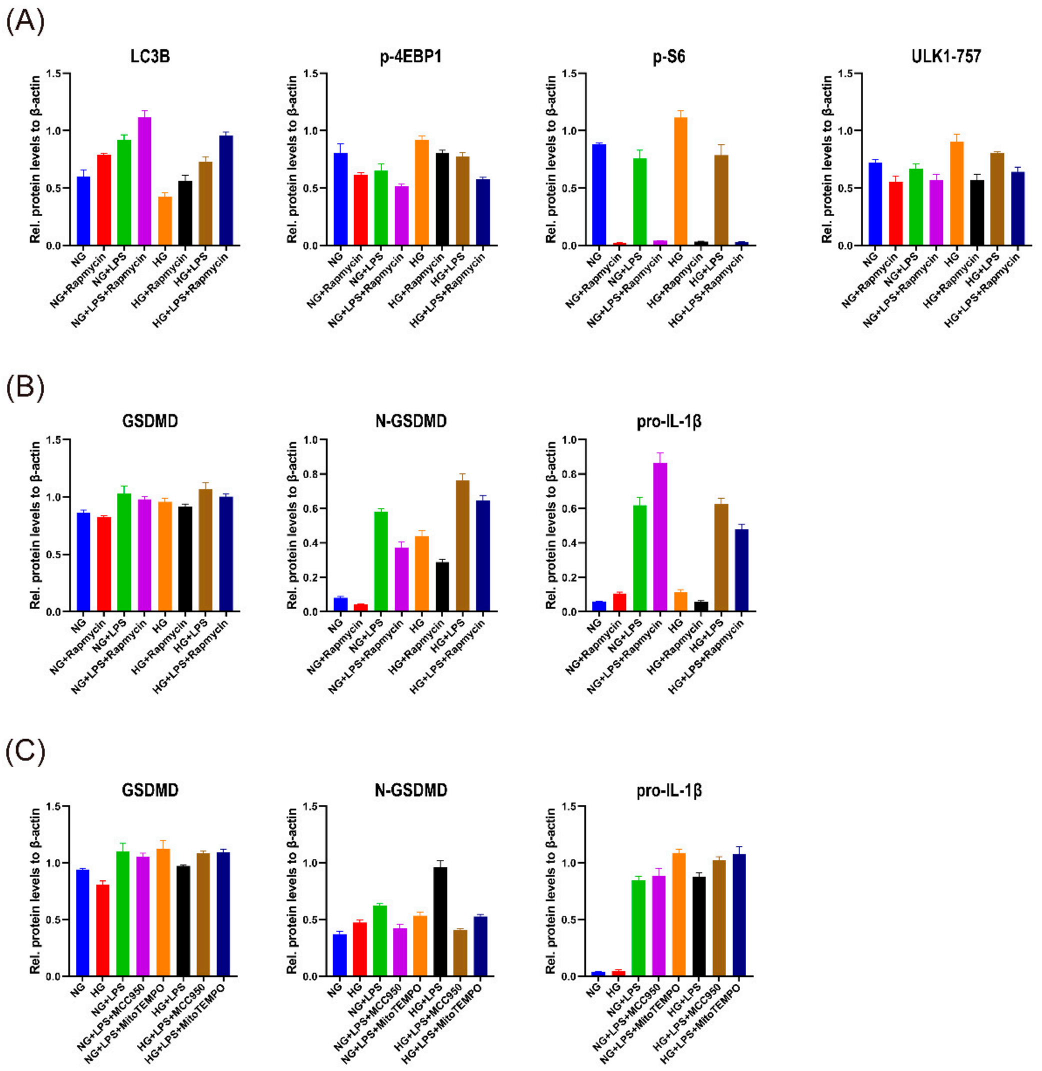

Figure S6. Quantitative analysis of the results of western blot in Figure 6A, B, E.

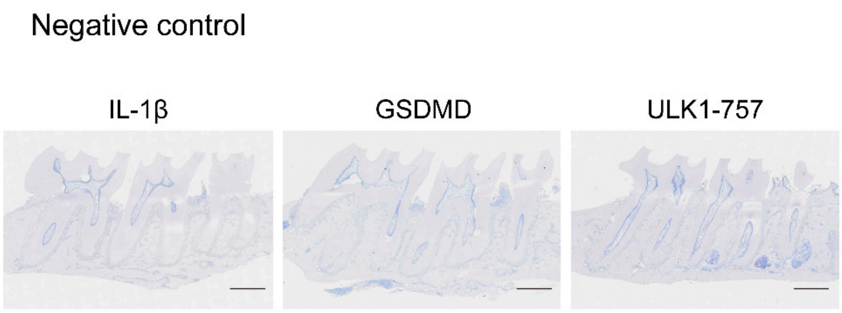

Figure S7. Negative control of IL-1β, GSDMD and ULK1-757 of immunohistochemistry staining (scale bar: 1mm).
